# Prevention of Postoperative Atrial Fibrillation - A Stitch in Time

**Published:** 2011-02-07

**Authors:** Raja Selvaraj

**Affiliations:** Department of Cardiology, Jawaharlal Institute of Post Graduate Medical Education and Research, Puducherry- 605006

**Keywords:** Atrial Fibrillation, Postoperative, Prevention

Atrial fibrillation (AF) is the most common arrhythmic complication in the postoperative period, occurring in about a third of patients undergoing coronary artery bypass graft surgery [1,2]. It is typically seen between the second and fourth postoperative days [3] and is associated with a significantly increased risk of postoperative stroke [1] and in-hospital mortality [4]. Treatment strategies are targeted at rate or rhythm control and anticoagulation for prevention of thromboembolic complications. However, treatment is often difficult and associated with significant morbidity, making this a classic situation where prevention is better than cure.

Pharmacological interventions have been the mainstay of preventive therapy. Beta blockers have been shown to be the most effective preventive therapy and it is recommended now to continue or initiate beta blocker therapy for all patients in the perioperative period [5]. Amiodarone is also an effective drug, but the intravenous preparation is associated with a risk of hypotension [6] and oral therapy has to be begun several days before surgery [7]. Sotalol [8], magnesium [9], statins [10,11], N-3 polyunsaturated fatty acids [12] and anti-inflammatory agents [13] are other pharmacologic measures that have been shown to be useful in various trials.

Atrial pacing is an attractive non-pharmacological intervention for the prevention of atrial fibrillation. Pacing in the postoperative period is easy to implement since it is only required for a short period and can therefore be performed using epicardial temporary pacing wires placed by the surgeon. The mechanisms by which atrial pacing is postulated to reduce the incidence of atrial fibrillation include reduction of bradycardia induced dispersion of atrial repolarization and overdrive suppression of atrial premature beats. Dual site atrial or biatrial pacing may result in additional benefit by promoting more synchronised atrial depolarization which results in reduced dispersion of atrial refractoriness and by altered atrial activation patterns that may prevent the development of intra-atrial reentry [14]. Prophylactic pacing has been shown in a number of trials to reduce the incidence of AF after CABG [15]. In a small randomized trial, biatrial pacing was shown to be superior to single site atrial pacing [16].

In this issue of the journal, Chavan et al [17] report on their results with the use of Bachmann bundle pacing as an alternative approach to pacing in the postoperative period. Significantly reduced paced P wave duration confirms the hypothesis that pacing at this site results in more synchronised atrial activation with lesser total atrial activation time. This, in turn, led to a significantly reduced incidence of AF compared to patients who received conventional right atrial pacing or those with no pacing. Although the results need to be interpreted with caution given the small number of study patients and a previous trial showing no benefit with atrial septal pacing [18], the results suggest promise for Bachmann bundle pacing to emerge as a simple preventive measure that may be at least as effective as pharmacologic therapy without the associated adverse effects.
